# Fatty kidney disease: The importance of ectopic fat deposition and the potential value of imaging

**DOI:** 10.1111/1753-0407.13232

**Published:** 2021-10-27

**Authors:** Christian Mende, Daniel Einhorn

**Affiliations:** ^1^ Medicine University of California at San Diego La Jolla California USA; ^2^ San Diego Endocrine Associates Scripps Whittier Diabetes Institute La Jolla California USA

The term “fatty kidney” first appeared in the literature in 1883^1^ and 100 years later it was suggested that hyperlipidemia is the cause of the characteristic renal lipid accumulation and nephrotoxicity.^2^ We now better understand the relationship between ectopic lipid accumulation in and around the kidney and the pathophysiologic consequences of visceral obesity. This review summarizes three clinically distinct renal consequences of visceral obesity and combine them into a distinct entity called fatty kidney disease (FKD).^3^

The prevalence of obesity, defined by the World Health Organization as a body mass index >30, is now over 42% in US adults.^4^ Visceral adiposity, a distinct subset of obesity often used interchangeably, is a well‐recognized risk factor for metabolic syndrome, prediabetes, diabetes, hypertension, cardiovascular disease, and heart failure.^5^, ^6^ Less well recognized is that obesity is associated with a 36% increase of chronic kidney disease (CKD), a 51% increased risk of new‐onset albuminuria, and a 3‐fold risk of developing end‐stage kidney disease.^7^, ^8^

We compare FKD to fatty liver disease, beginning with non‐alcoholic fatty liver disease (NAFLD) and progressing to non‐alcoholic steatohepatitis (NASH). NAFLD has been recognized as a risk for albuminuria, CKD, and its progression, but a specific type of renal involvement or renal pathology has not been described as there are no renal biopsy reports in studies of both NAFLD and CKD combined. As delineated by Byrne it is the many risk factors that are shared by both diseases that would be the likely cause of their frequent co‐existence.^9^

Visceral adipose tissue (VAT) may have broad pathological consequences that are different in different organs. For example, there may be differences in the threshold for how much VAT exists and when a consequence is manifest in a given organ. Perhaps FKD will be found to occur whenever NAFLD occurs, in which case its presence could be inferred without being imaged separately.

We realize that there are no currently established criteria for the radiological diagnosis of FKD. However, the extensive renal sinus fat (RSF) and perirenal fat accumulations that are routinely seen on magnetic resonance imaging (MRI) or computed tomography (CT) are seldom even mentioned in reports, despite being clinically relevant as risk factors for hypertension and CKD.^10^, ^11^ We argue that they deserve to be recognized and documented in radiological reports. This echoes the lack of reporting on liver fat 25 years ago.

As the understanding of NAFLD unfolded, radiologic imaging played a critical role in defining the presence, severity, and evolution of the disease. We encourage radiologists to do the same for FKD in order to enhance our knowledge of how ectopic fat contributes to FKD. It is possible that FKD routinely accompanies NAFLD and ectopic fat in other organs to produce the organ‐specific and systemic manifestations of diabetes and metabolic syndrome.

## 1 INTRODUCTION

Free fatty acids (FFA) and other lipids have important normal physiologic functions, serving as a source for energy, for cell wall components, for intra‐ and extracellular signaling, and more. For example, FFA carried by albumin to the kidney is a major source of ATP production via beta oxidation in the proximal tubule.^12^

FFA plasma levels are normally between 0.2 to 0.6 mM, but can be elevated 10‐fold in obesity, diabetes, metabolic syndrome, NAFLD, and other conditions.^13^ Excess caloric intake leads to de novo lipogenesis and storage of FFA, triglycerides (TG), and other lipids by the adipocytes, the only cell type specialized to do so. Adipocytes can increase their lipid storage capacity by hypertrophy and hyperplasia but only to a limited extent. When that capacity is exceeded, FFA rise rapidly in the circulation. Elevated FFA levels lead to ectopic fat deposits in multiple organs and that in turn leads to lipotoxicity.^14^

Lipotoxicity has multiple components. It involves conversion of FFA and TG into diacylglycerol and ceramide, thereby interfering with lipogenic and glycogenic cell‐signaling pathways.^15^ Infiltration of ectopic adipose tissue by macrophages initiates an inflammatory milieu with increased release of cytokines, including tumor necrosis factor (TNF) alpha, interleukin (IL) 1 and IL 6 with elevated human C‐reactive protein levels.^16^ Lipotoxicity also causes mitochondrial dysfunction, leading to production of reactive oxygen species, inflammation, and apoptosis.^15^

Organ‐specific ectopic fat deposition has been described most thoroughly for NAFLD and NASH. However, ectopic fat deposits occur in multiple organs, such as skeletal muscle. In the epicardium, ectopic fat deposits located between the pericardium and myocardium may be mediators of risk for coronary artery disease, heart failure, and atrial fibrillation. Epicardial fat thickness, measured by ultrasound, correlates with severity of coronary artery disease and interferes with left ventricular function.^17^ This contributes to diastolic dysfunction and creates a source of inflammatory cytokines.^18^

Pancreatic fat deposits are increased in prediabetes and diabetes, but without known direct relation to beta cell function.^19^ A metanalysis of pancreatic fat content measured by MRI revealed that increased pancreatic fat was associated with a 2‐fold risk of metabolic syndrome and type 2 diabetes.^20^ The literature remains unclear, however, regarding the specific influence of ectopic pancreatic fat on beta cell function.

In the kidney, ectopic fat deposits produce FKD. Many effects of the individual components of visceral obesity on the kidney have been well described previously but not in a unifying fashion that this paper proposes to accomplish. Focal intracellular lipid vacuoles accumulated in mesangial cells, podocytes, and proximal tubular epithelial cells positive oil‐red‐o‐staining) have been noted in obesity‐related glomerulopathy (ORG).^12^, ^21^ Renal biopsies show cholesterol deposits in some causes of CKD.^22^, ^23^

FKD may be considered a three‐part disorder wherein obesity leads to three distinct clinical presentations, each with a different impact on the kidney and on systemic manifestations. We describe FKD in a pathophysiologic manner as a new entity encompassing three distinct elements:

1. The effects of **intraabdominal fat accumulation** on the kidney. This includes hyperfiltration, glomerular hypertension, albuminuria, renal insulin resistance, and release of proinflammatory cytokines.
2. The effects of **perirenal ectopic fat**, especially **hilar/RSF** with (a) physical compression of vascular and nerve bundle, upregulation of the renin–angiotensin–aldosterone system (RAAS) and sympathetic nervous system and increased tubular sodium reabsorption and (b) local release of cytokines
3. **Parenchymal ectopic fat** deposits

### 1.1 Effects of visceral obesity on the kidney overall

The overall milieu caused by visceral obesity must be taken into account as a component of FKD. Obesity itself is associated with increased renal tubular sodium reabsorption followed by initial hyperfiltration and subsequent gradual decline of estimated glomerular filtration rate (eGFR).^24^ Adipocytes are able to secret all components of the RAAS and are found to be upregulated in obesity.^25^

Aldosterone levels are elevated in obesity secondary to higher angiotensin II, leptin, and aldosterone synthase.^26^ In addition, the high salt intake that typically accompanies excessive caloric intake upregulates the mineralocorticoid receptor contributing to podocyte injury, albuminuria, CKD, and hypertension.^27^

An example of the unique impact of obesity on the kidney is ORG, a seldom seen component of FKD, but a well‐described entity in which both visceral fat deposits and renal parenchymal fat droplets in the podocytes and tubular and mesangial cells are seen^21^, ^28^ The histological components of ORG include glomerular cell proliferation, glomerulomegaly, thickening of basement membrane, increased mesangial matrix deposition, loss of podocytes, and focal glomerulosclerosis. Clinical presentations are isolated albuminuria as high as nephrotic range and development of progressive CKD.^28^

The mechanisms underlying ORG are not fully understood but appear to reflect the kidneyʼs unique response to visceral obesity. They include hemodynamic and inflammatory factors, activation of the RAAS, increased sympathetic nervous system activity, elevated leptin, and lower adipokine levels.^21^

Although ORG and the associated pathophysiology have been described separately before, together they represent one part of the overall milieu created by visceral obesity as a component of FKD.

### 1.2 Effects of ectopic fat deposits in the perirenal space, the hilum, and the sinus

Deposition of fatty tissue occurs in several areas of the kidney, including the retroperitoneal space, the perinephric space outside the renal capsule, the hilum, and the sinus area. All of these can contribute to direct physical compression of the kidney, interfering with renal function.

Compression may be compounded by elevated intra‐abdominal pressures, measured as high as 40 mm Hg^29^ This physical pressure on the kidney causes stimulation of the RAAS and increases renal tubular sodium reabsorption and the risk of hypertension.^24^ The renal hilum is especially sensitive to pressure as it is not covered by the renal capsule, thus allowing the elevated pressures to be directly transmitted to the medulla.^11^, ^29^

**FIGURE 1:**
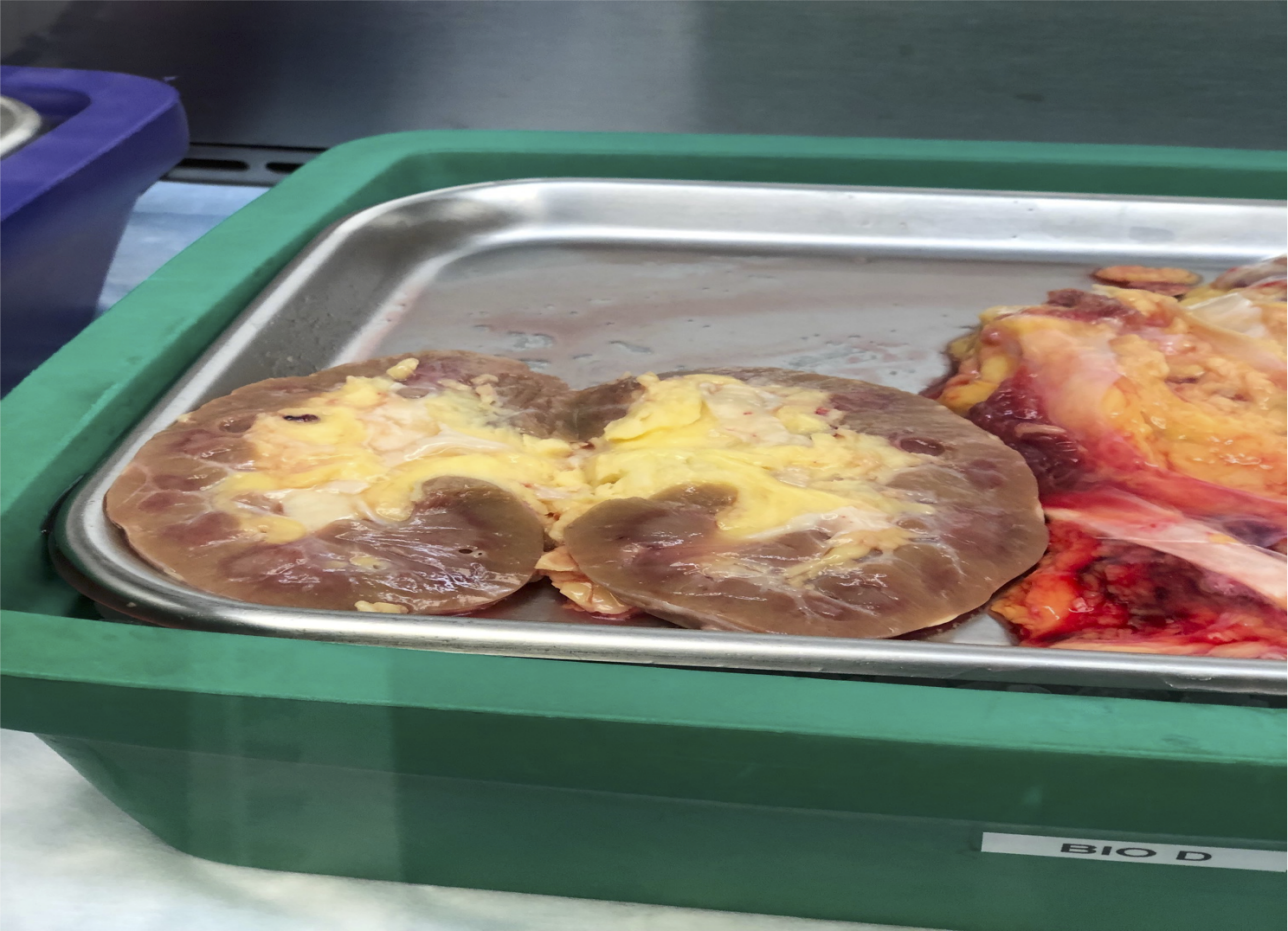
Autopsy specimen of fatty kidney

Because of the absence of the renal capsule at the hilum, fat accumulation around the hilum and sinus directly compresses arterial and venous blood flow as well as the nerve bundle, reducing arterial inflow and increasing intrarenal venous pressure with a risk of reducing eGFR. The lack of a hilar capsule also allows extrarenal fat to readily extend into the renal medullar space, as shown graphically in a human kidney autopsy specimen from an obese patient (Figure 1).

The mechanical pressure and the expansion of RSF result in hypoperfusion of renal parenchyma and compression of tubules with reduced flow rates.^30^ As a consequence of the resulting local ischemia, elevated levels of serum kidney injury molecule‐1 (sKIM‐1), a marker of renal tubular injury, are seen.^31^ KIM‐1 has been directly correlated with the volume of RSF.^32^ At the loop of Henle, this results in stimulation of renin release with increased formation of angiotensin II and fractional sodium reabsorption.^24^

RSF volume, measured by MRI, is associated with increased renal vascular resistance in obese diabetic patients with normal renal function.^30^ Increased renal vascular resistance can reduce eGFR and is a risk factor for CKD.^31^ RSF is significantly increased in prediabetic and diabetic patients and correlated with visceral fat deposition.^32^ RSF may act in a paracrine fashion, secreting proinflammatory cytokines that negatively affect renal function.^33^ RSF has been postulated to be a link between metabolic disease and CKD.^32^

In type 2 diabetes the perirenal fat thickness has been shown to be an independent predictor of renal dysfunction, loss of eGFR, increased renal resistance, and hyperuricemia.^34^ Ultrasound evaluation of perirenal fat thickness predicted early kidney injury in obesity.^35^

“Perirenal adipose tissue is a complex microenvironment … contributing in the pathogenesis of hypertension, obesity, chronic renal diseases…(acting) as an endocrine organ”.^36^ In the Framingham Heart Study renal CT showed renal sinus fat deposits in about 30% of the participants, which in a subsequent 10‐year follow‐up was associated with more than doubling of new onset of CKD and hypertension.^10^

### 1.3 Effects of renal parenchymal fat deposits

Ectopic intrarenal parenchymal lipid deposits cause lipotoxicity with albuminuria and CKD and also have systemic effects.^37^ Cellular uptake of lipids from the circulation in the form of FFA, TG, or cholesterol is facilitated by fatty acid transport proteins and other transporters^37^, ^38^ and are stored as tiny lipid droplets (20‐40 nm). This occurs in the kidney in multiple cells, including podocytes and proximal tubular epithelium and mesangial cells.^38^

Other factors contributing to renal parenchymal lipid deposits include hypertriglyceridemia and hyperglycemia itself, by increasing lipid synthesis and TG accumulation.^39^ The resulting excessive lipid accumulation may contribute to formation of toxic metabolites such as ceramide and diacylglyceride that in turn may interfere with cell function. Many of the mediators of this lipotoxicity have been identified, including TNF alpha and IL 6.^15^ These are known to cause oxidative stress, mitochondrial dysfunction, apoptosis, inflammation, and fibrosis.

FFA accumulation in podocytes causes local insulin resistance and apoptosis.^38^, ^40^, ^41^ These have been described in diabetic kidney disease, focal glomerulosclerosis, Fabryʼs disease, and hypertensive nephrosclerosis and are part of the spectrum of FKD.^21^ Podocyte lipotoxicity resulting from the accumulation of FFA is the major cause of the initiation and progression of albuminuria and CKD via impaired insulin signaling.^38^ In addition, mitochondrial dysfunction, with reduced availability of ATP, results in apoptosis.^23^ FFA stimulate renal and hepatic gluconeogenesis and compete with glucose use, thereby contributing to hyperglycemia in metabolic syndrome and diabetes.^42^

In summary, the numerous effects of intrarenal parenchymal fat deposits create both damage to the kidney and systemic effects contributing to metabolic syndrome.

## 2 RECOGNIZING FKD CLINICALLY ‐ THE NEED FOR RADIOGRAPHIC IMAGING

NASH and NAFLD were not reported on abdominal imaging until their clinical importance was recognized. Since then, imaging characteristics of NASH and NAFLD have helped to develop the understanding of fatty liver disease and have led to important innovations.

FKD is currently in the same situation that NASH and NAFLD were 20 years ago. Radiologic reports of MRI and CT scans of the abdomen typically do not elaborate on the presence of ectopic renal hilar or sinus fat, presumably being unaware of its clinical significance. It would help the understanding and recognition of FKD as a distinct entity if radiologists can help define the imaging criteria for FKD.

Currently, renal cortical fat deposits are difficult to measure except with Proton MRI. There are issues of the boundary of the renal capsule and perirenal fat and the paucity of intrarenal fat normally. Advances in renal ultrasound, CT, and MRI may be able to overcome these obstacles and allow a radiographic diagnosis and better characterization.

MRI and CT are presently able to delineate only perirenal fat, hilar fat, and RSF. Hilar fat and RSF should perhaps be considered a combined “entity” because a demarcation is not possible. The assessment of renal triglyceride content by Dutch groups is encouraging.^43^, ^44^

Noninvasive molecular imaging of kidney disease is progressing and, it is hoped, can measure renal triglyceride/fat as well.^45^, ^46^

In a weight loss study, MRI was able to assess fat loss in RSF but could not detect any change in renal parenchymal fat.^47^

Ultrasound elastography of the kidney may be a technique to measure renal fat content.^48^ Sonographic evaluation of para‐ and perirenal fat thickness has been used as a predictor of early kidney damage in obese patients.^35^ B‐mode renal ultrasound targeting perirenal fat thickness in specific locations is showing promise.^49^ “Para and peri‐renal fat ultrasonographic thickness may be … a useful tool for the assessment of visceral fat and early kidney damage in obese adults”.^35^

Much might be learned from such imaging and some centers are beginning the radiographic exploration of FKD.^43^, ^46^

Because visceral obesity affects multiple organs, it is plausible that FKD occurs whenever NAFLD occurs, as suggested by human^50^, ^51^ and animal studies.^46^

If this is confirmed by imaging studies, it may be possible to infer the presence of FKD whenever NASH is identified, thus obviating the need for specific renal imaging. To the extent that imaging can quantify the degree and location of ectopic fat, there may a quantifiable threshold of visceral obesity that initiates the pathological consequences in the kidney as it appears to do in the liver.

Similarly, there may be implications for recognizing that the location and amount of fat accumulation in other organs, such as the epicardium, determines the extent of pathological consequences, both systemically and to the organ itself.

Pig data show a 100% correlation between the presence of NAFLD and fatty kidney by renal biopsies and triglyceride measured by MRI 7‐Tesla.^46^ We postulate that this coexistence also is present in humans, mainly because of many parallel associated factors (metabolic syndrome, insulin resistance, dyslipidemia, proinflammatory state, etc.). No specific type of renal disease has been described when NAFLD and CKD are both present as there are no data published on renal biopsies in that setting. No causal link between NAFLD and CKD has been established, but the relationship has been reviewed in detail.^9^

It is certainly possible that the time course and nature of progression of NAFLD to NASH differs from that of FKD to CKD, but they both would be expected to reflect the toxicity of ectopic fat deposits.

FKD (ie, all three stated components: ORG, hilar/renal sinus fat, and intrarenal ectopic fat deposits) has been shown to be a major risk factor for CKD initiation and progression for CKD and hypertension in multiple publications.^7^, ^10^, ^11^, ^24^

One key feature of renal cortical lipid deposition (triglyceride and lipid droplets) is podocyte toxicity with albuminuria and CKD development.^23^

## 3 THE IMPORTANCE OF FATTY KIDNEY DISEASE

In and of itself, the concept of FKD, consisting of three distinct clinical components, helps us understand the role of the kidney in contributing to the pathophysiology of diabetes and the cardiorenal consequences.

It also advances the unifying concept of visceral obesity as a multiorgan systemic disease that has many opportunities for prevention and treatment. FKD recognizes that the kidney is not just a victim but rather an active co‐conspirator in diabetes and metabolic syndrome.

## CONFLICT OF INTERESTS

The authors declare no potential conflict of interest.
